# Successive Immunization With Epitope-Decreasing Dengue Antigens Induced Conservative Anti-Dengue Immune Responses

**DOI:** 10.3389/fimmu.2020.585133

**Published:** 2020-09-25

**Authors:** Jue Hou, Weijian Ye, Hooi Linn Loo, Lan Hiong Wong, Jianzhu Chen

**Affiliations:** ^1^Interdisciplinary Research Group in Infectious Diseases, Singapore-MIT Alliance for Research and Technology (SMART), Singapore, Singapore; ^2^Koch Institute for Integrative Cancer Research and Departments of Biology, Massachusetts Institute of Technology, Cambridge, MA, United States

**Keywords:** dengue virus, vaccine development, sequential immunization, immunoglobulin diversification, epitope-decreasing

## Abstract

Repeated homologous antigen immunization has been hypothesized to hinder antibody diversification, whereas sequential immunization with heterologous immunogens can educate B cell differentiations towards conserved residues thereby facilitating the generation of cross-reactive immunity. In this study, we developed a sequential vaccination strategy that utilized epitope-decreasing antigens to reinforce the cross-reactivity of T and B cell immune responses against all four serotypes dengue virus. The epitope-decreasing immunization was implemented by sequentially inoculating mice with antigens of decreasing domain complexity that first immunized with DENV1 live-attenuated virus, following by the Envelope protein (Env), and then Env domain III (EDIII) subunit protein. When compared to mice immunized with DENV1 live-attenuated virus three times, epitope-decreasing immunization induced higher TNF-α CD8^+^ T cell immune response against consensus epitopes. Epitope-decreasing immunization also significantly improved neutralizing antibody response to heterologous serotypes. Moreover, this sequential approach promoted somatic hypermutations in the immunoglobulin gene of antigen-specific memory B cells in comparison to repeated immunization. This proof-of-concept work on epitope-decreasing sequential vaccination sheds light on how successively exposing the immune system to decreasing-epitope antigens can better induce cross-reactive antibodies.

## Introduction

A major challenge in dengue vaccine development is to induce robust and protective cross-reactive immunity against all four serotypes of dengue viruses (DENV). DENV 1–4 are antigenically distinct, but closely related viruses (1, 2) with up to 70% sequence homology (3, 4). In endemic countries, co-circulation of multiple serotypes of DENV is prevalent, and therefore, chances of getting either co-infection with multiple serotypes or sequential encounter with heterotypic dengue viruses is a common phenomenon (5).

The current licensed DENV vaccine, Dengvaxia, is a tetravalent vaccine composed of four DENV serotypes. However, the vaccine has limited overall efficacy (approx. 60%) against acute dengue, with only 50% and 35–42% for DENV1 and DENV2, respectively (6–8). Besides antigen selection, optimization and vaccine types in vaccine development, the immunization regimen is also critical. Traditionally, vaccination regimens employ multiple inoculations of immunogens, such as inactivated virus and cocktails of antigens. However, such regimens might not be optimal. Conserved epitopes may be masked by highly variable regions (9, 10), and the presence of these more accessible and non-immunogenic epitopes can “distract” B cell responses (11). Indeed, in a monkey DENV vaccination model, interference between DENV serotypes and immunodominance of certain epitopes led to dominance of neutralizing antibody titers against DENV4 (12). Instead, sequential immunization with a series of directional immunogens with decreasing epitope modifications have been shown to elicit heterologous neutralizing responses against HIV-1 (13). Additionally, our recent publication clearly demonstrated sequential immunization induced stronger and broader T and B immunity against four DENV serotypes than tetravalent-formulated immunization (14). The underlying mechanism of this strategy relies on directing B cell education. The successive boosting with epitopes of decreasing complexity forced B cells to rearrange the immunoglobin and promoted antibody avidity to recognize the conserved epitopes through somatic hypermutations.

One concern for sequential vaccination in DENV vaccine development is the phenomenon of antibody-dependent enhancement (ADE) (15–17). The ADE hypothesis suggests that pre-existing antibodies generated in response to a primary infection may have insufficient antibody avidity or concentration to neutralize secondary infection by a different dengue serotype. During secondary dengue infection, such weakly neutralizing antibodies may promote the infection of Fc receptor-bearing cells leading to virus amplification, cytokine storm and subsequent plasma leakage (18). The occurrence of ADE thus raises safety concerns about whether incomplete protection against all four dengue serotypes prior to complete vaccination can increase disease severity (19). The results from a phase 2b trial of CYD tetravalent dengue vaccine in Thai schoolchildren requiring multiple vaccination doses showed no increased risk for severe diseases during the course of the vaccinations (6). Consistently, clinical trials on monovalent chimeric dengue vaccine (20) and bivalent CYD dengue vaccine (21) both showed a lack of adverse events and viremia after heterotypic dengue vaccine inoculation. These findings demonstrated the safety and feasibility of sequential vaccination.

In this study, we examine whether introducing a series of directional DENV immunogens with decreasing epitope modifications sequentially can improve both T cell and B cell immune responses against four DENV serotypes in mice model. Our results show that epitope-decreasing vaccination potentially induces higher cellular immune responses targeting conserved epitopes compared to repeated immunization with a priming immunogen. We further study the immunoglobulin diversification in antigen-specific B cells after each immunization to understand the evolutionary dynamics in different immunization approaches.

## Materials and Methods

### Mice and Immunization Regimens

C57BL/6J (B6) mice were used for the experiment. Mice were bred and housed at the Animal Facility, National University of Singapore (NUS). All procedures and care were approved by the NUS Research Ethics Committee under Protocol R13-6157. All ethical regulations regarding animal research were complied with.

**Table 1** depicts the immunization schedules of two vaccination strategies. For repeated immunization, 1×10^6 PFU Dengue 1 live virus (strain 2402DK1) (DENV1) in 50 µl volume was used per injection, repeated three times. For epitope-decreasing immunization, 1×10^6 PFU DENV1/50 µl, 10 µg/50 µl DENV1 extracellular domain Envelope protein (Env) (CTK Biotech), and 10 µg/50 µl DENV1 Env protein domain III (EDIII, in-house production) were administered at the first, second, and third dose, respectively. Two groups of 8-week old female B6 mice (5 mice per group) were immunized 3 times intramuscularly with 2 weeks apart each dose under general anesthesia. Two weeks after the final dose, the mice were sacrificed for terminal analysis.

**Table 1:** Immunization schemes.

Group	Immunization	1^st^ shot	2^nd^ shot	3^rd^ shot
1	Repeated	DENV1	DENV1	DENV1
2	Epitope-decreased	DENV1	DENV1/Env	DENV1/EDIII

### Intracellular Cytokine Staining

Splenocytes from immunized mice was assessed for cytokine production by intracellular cytokine staining as described previously (22). Briefly, 1 million splenocytes were stimulated with a peptide cocktail (23) or each serotype virus (DENV1/2402DK1, DENV2/3295DK1, DENV3/863DK1 and DENV4/2240DK1). Cells were surface stained with anti-CD4 and -CD8 et al. primary antibodies followed by intracellular staining with anti-TNFα monoclonal antibodies. Data were acquired on LSRII flow cytometer (BD Biosciences) and analyzed using FlowJo (version 10.6.0 Tree Star).

### Dengue Plaque Reduction Neutralization Test (PRNT)

Neutralizing antibody titer (nAb) was determined by PRNT as previously described (24) on four strains DENV1/2402DK1, DENV2/3295DK1, DENV3/863DK1 and DENV4/2240DK1. The highest serum dilution that resulted in 50% or more plaques reduction compared to the virus control wells was considered as the neutralizing endpoint titer (PRNT_50_).

### EDIII-Specific Binding Antibody ELISA Assay

Ninety-six-well plates were coated with 1µg/ml in-house produced recombinant EDIII protein and kept at 4°C overnight. The plates were washed 5 times with PBST (0.05% Tween 20) and blocked with 5% BSA at 4°C overnight. After washing, serum samples were added to plates in dilution from 1:200 to 1:25,600 and incubated for 2 h in 37°C. Secondary HRP-labeled anti-mouse IgG diluted to 1:5000 was added to plates and incubated for 1 h at 37°C. TMB substrate was added and the absorbance was read at 450nm. The cut-off threshold was set at least two times higher than the result of negative sera sample. The titer was determined by the last dilution giving value above the cut-off threshold.

### B Cell Assays

The antigen-specific B cell responses were probed by fluorochromes labeled DENV1 and DENV2 E proteins as previously described (24). Cell were analyzed on an X20 flow cytometer (BD Biosciences) and data processed using FlowJo version 10.6.0 (Tree Star).

### Antigen-Specific Immunoglobulin Repertoire Sequencing by RNA-Seq

A total of 10,000 DENV-specific B cells (either DENV1^+^ or DENV2^+^ or DENV1^+^DENV2^+^) were sorted on FACS Aria II cell sorter (BD Biosciences). The RNA extraction, cDNA synthesis and target gene amplification, sequencing library preparation as described previously (14). The libraries were multiplexed and subjected to MiSeq V3 2×301 bp sequencing.

Raw sequences were processed using the toolkit “pRESTO” (version 0.5.13) (25). Briefly, the paired-ends MiSeq data was firstly assembled into a full-length B cell receptor (BCR) sequences, followed by removing the low-quality reads, annotating Ig isotype, masking the primer regions and yielding the final sequences comprised of unique sequence with at least two representative reads. The IMGT/High database of mouse immunoglobulin repertoire was used as reference to perform V(D)J alignment using IgBLAST in tool “Change-O” (version 0.4.6) (26). The V segment genotypes were inferred using package “TIgGER” (version 0.2.10) (26). Ig sequences were assigned into clonally related lineages and the full germline sequences were built after preforming automated detection of the clonal assignment threshold by using package “SHazaM” (version 0.2.1) (26). Mutations were defined as nucleotides that were different from the inferred germline sequence. The clonal diversity of the repertoire was analyzed using the general form of the diversity index, as proposed by Hill (27) and implemented in the package “Alakazam” (version 0.3.0) (26). The somatic hypermutation targeting models were computed by the SHazaM software (version 0.2.1) (26). The raw data has been deposited in Gene Expression Omnibus (GSE154371).

### Statistical Analysis

The statistical analysis of T and B cell responses and nAb titer were performed using two-sided Mann-Whitney test in GraphPad Prism 7.0 software (GraphPad Software Inc.). The statistical comparisons between strategies at indicated doses on Ig repertories mutation frequency were calculated using unpaired two-sided Wilcoxon test in R.

## Results

### Epitope-Decreasing Sequential Immunization Induced Potent T Cell Response to Conserved Epitopes

**Figure 1:**
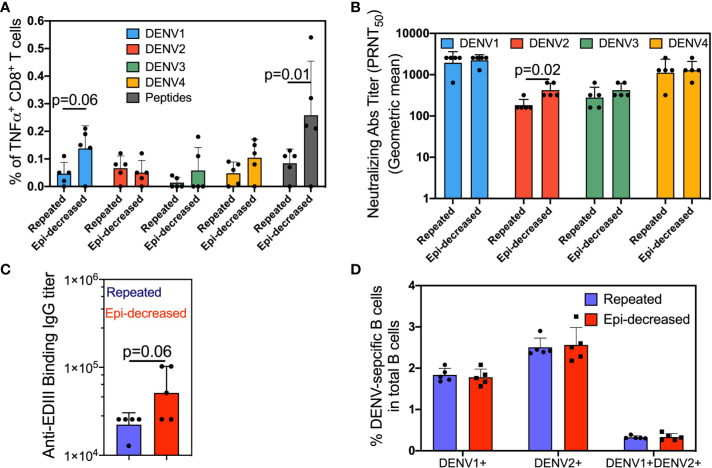
The DENV-specific cellular and humoral immune responses. **(A)** Splenocytes were stimulated with each of the four DENV (DENV1/2402DK1, DENV2/3295DK1, DENV3/863DK1 and DENV4/2240DK1, at M.O.I = 1) or consensus envelope and capsid peptide pool (at a final concentration 5 µg/ml per peptide) or medium as control for 6 h at 37°C in the presence of BFA. Cells were surface stained for CD3 and CD8, and intracellularly stained for TNFα. The plots show the percentage of TNFα producing CD8^+^ T cells. Each dot represents one mouse. The bar plots show the mean value and SD, and the colors indicate antigens used for stimulation. The p value denotes the comparison results between the indicated comparisons were calculated by Mann-Whitney test. **(B)** The neutralization antibody titers two weeks after the 3^rd^ immunization were measured by plaque reduction neutralization test (PRNT) assay. Four serotypes of dengue virus were separately incubated with serially diluted sera to measure the neutralization capability of reactive antibodies. The serotype specific neutralizing antibodies were determined by 50% plaques reduction compared to the virus control wells. The data shows as geometric mean titers ± geometric SD in the bar plot. Each dot represents one mouse. The p values between the indicated comparisons were calculated by Mann-Whitney test. **(C)** Anti-DENV Env EDIII Ig binding Ab titers in the sera after the 3rd immunization specific were measured by ELISA. The data shown are geometric mean titers ± geometric SD. Each dot represents one mouse. The p value shows the Mann-Whitney comparison result between two groups. **(D)** Two weeks after last immunization, splenocytes were stained with Alexa conjugated DENV1 and DENV2 E proteins and appropriate antibodies. The antigen specific DENV1^+^, DENV2^+^ and DENV1^+^DENV2^+^ B cells were assessed. The bar plots show the mean value and SD. Each dot represents one mouse. The p values were calculated by Mann-Whitney test.

We compared the T cell immune responses between mice that were immunized with three doses of live DENV1 virus (repeated immunization) and mice that were sequentially immunized with live DENV1 virus, DENV1 Env protein and DENV1 EDIII subunit protein (epitope-decreasing immunization). Two weeks following the last immunization, splenocytes were harvested and stimulated with a mixture of either consensus DENV peptides or DENV1-4 and analyzed CD8^+^ T cells for TNFα production. In contrast to repeated immunization, epitope-decreasing immunization induced higher level of both homotypic (DENV1) and heterotypic (DENV3/DENV4) specific TNFα-producing CD8^+^ T cells (**Figure 1A**). Importantly, compared with repeated immunization, epitope-decreasing immunization reinforced antigen-specific CD8^+^ T cells responding to consensus DENV peptides stimulation (**Figure 1A**). This indicated that epitope-decreasing immunization potentially narrowed down T cell response to specific and conservative epitopes.

Next, we compared two groups on the neutralizing antibody (nAb) titer and anti-EDIII binding antibody titer in serum samples of immunized mice after 3 doses. As expected, both immunization regimens elicited strong homologous nAb responses against DENV1. Heterotypic nAb responses against DENV2, DENV3, and DENV4 were also observed, although at lower titers compared to anti-DENV1 (**Figure 1B**). Notably, epitope-decreasing immunization significantly boosted anti-DENV2 nAb response to near 2 times than the titer obtained from repeated immunization. Moreover, binding ELISA assay demonstrated epitope-decreasing immunization induced higher IgG titer against the conserved EDIII domain compared to repeated immunization (**Figure 1C**) with near significance.

To further investigate and quantify homo- and heterotypic antigen-specific B cell responses following two immunizations, we utilized DENV1/E-AF647 and DENV2/E-AF548 to stain for DENV1 and DENV2-specific B cells in the spleen two weeks after the last immunization dose. Both repeated immunization and epitope-decreasing immunization induced comparable levels of homotypic DENV1^+^ or heterotypic DENV2^+^ single positive, and cross-reactive (DENV1^+^ and DENV2^+^ double-positive) B cells (**Figure 1D**).

Cumulatively, these results suggest that epitope-decreasing immunization strategy is beneficial for inducing T cell responses and promoting nAb responses that target conserved immunodominant regions, such as the EDIII domain.

### Epitope-Decreasing Sequential Immunization Promoted Immunoglobulin Mutation Frequency

**Figure 2:**
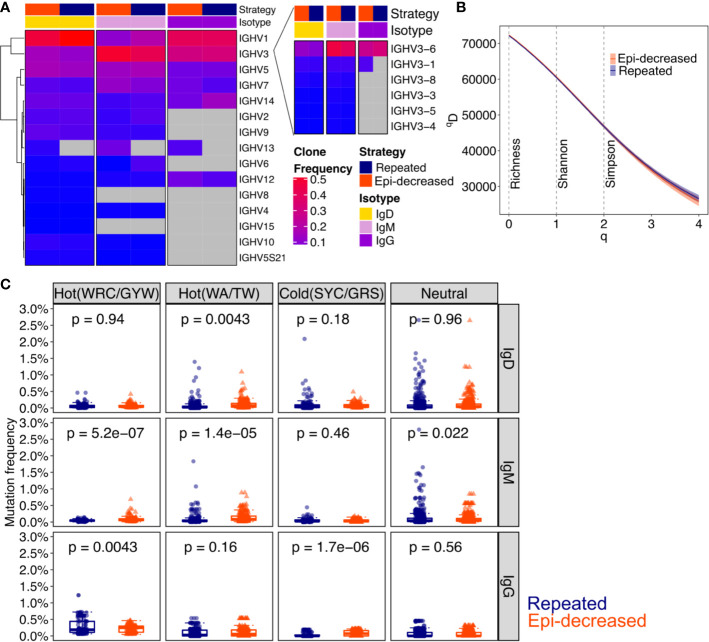
The characteristic of immunoglobulin and somatic hypermutation induced by different strategies. **(A)** Heatmap shows the V_H_ family usages by clone frequencies with red to blue to grey corresponding to from high, low to absence. Strategy indicates the immunization and isotype indicates IgD, IgM and IgG. The Chi-square test was performed for statistical analysis. **(B)** The clonal diversity analysis was performed by using the generalized Hill’s diversity index. The diversity index (^q^D) was calculated over a range of diversity orders (q) and plotted as a smooth curve. The ^q^D values depict the level of diversity for a given value of q. The lower ^q^D values represent lower diversity. Shaded area represents 95% percentiles. The Richness diversity index, which equates to q = 0, the Shannon diversity index, q = 1, and the Simpson diversity index, q = 2, were plotted as dashed vertical lines. **(C)** The bar plots for the levels of somatic hypermutation (SMH) in hot- and cold spots with Ig isotypes. SMH targeting profiles were analyzed for 5-mer motifs from both immunization strategies. The WRC/GYW hotspot motifs, WA/TW hotspot motifs, SYC/GRS cold spot motifs and neutral spots are shown. Each dot represents a 5-mer motif and each box covers the 25^th^ – 75^th^ percentiles of the mutability rates of the 5-mer motifs in its corresponding groups, with the horizontal bar indicating the median. The p values show the statistical significance by Wilcoxon test analysis for indicated groups.

To compare the diversity of immunoglobulin (Ig) repertoires between repeated and epitope-decreasing immunizations, we performed Ig-RNA sequencing on sorted DENV1^+^ and/or DENV2^+^ B cells. The sequencing details are described in **Table 2**. As DENV-specific B cells differentiated from IgM^+^/IgD^+^ to IgG^+^, the Ig heavy chain variable gene usage decreased. The IgG^+^ B cells predominantly used immunoglobulin heavy chain variable region genes (IGHV) 1, 3, 5, 7, and 14. Distinctively, IGHV13 (specifically, IGHV13-2) was only induced in epitope-decreasing immunization strategy. Based on the clone frequencies, the IGHV3-1 usage in IgG isotype was more prominent following epitope-decreasing immunization. IGHV3-6 usage was elevated in DENV^+^ B cells that expressed either IgD or IgM within epitope-decreasing immunization strategy (**Figure 2A**). Interestingly, the Ig diversification induced by each immunization was comparable (**Figure 2B**).

**Table 2:** The summary of RNA-seq results.

Group	Original Sequences	Assembled Sequences	Filtered Sequences	IgBlastClones	Final Repertoire
Repeated	2,926,869	2,764,584	123,356	30,758	28,134
Epi-decreasing	2,705,628	2,557,028	121,402	30,270	27,133

Somatic hypermutations (SHM) analysis revealed that repeated immunization boosted IgG WRC/GYW hot spot mutation, whereas epitope-decreasing immunization promoted IgG SYC/GRS cold spot mutation. Additionally, epitope-decreasing sequential immunization induced higher WRC/GYW and WA/TW hot spots mutation frequencies than repeated immunization in IgM isotype (**Figure 2C**).

Together, these results suggest that epitope-decreasing immunization, through antigen-driven progression, reinforces some mutations through somatic hypermutations to generate high specificity and affinity antibody that recognize conserved domains.

## Discussion

A comprehensive understanding of how vaccine elicits protective and broadly cross-reactive immune responses is critical when handling pathogens that deceive and escape the immune memory by continually changing their antigenic characteristics. Here, we investigate the effect of epitope-decreasing sequential immunization strategy on the generation of cross-reactive responses against DENV1-4. Epitope-decreasing immunization can be considered as a form of supervised learning, which guides the immune response to focus on conserved domains.

As neutralization of DENV1 is generally weaker compared to other dengue serotype, we investigated the T cell and B cell responses induced by repeated live DENV1 virus vaccination compared to sequential vaccination with DENV1 live virus, followed by DENV1 Env protein and finally by DENV1 EDIII subunit protein in mice model. In agreement with previous reports, as the complexity of the immunogens decreased, the immune responses elicited were guided toward immunodominant targets (13). In this study we show that epitope-decreasing immunization can reinforce specific T cell immune responses on consensus epitopes. Additionally, it also induces heterotypic humoral immunity as shown by nAb capable of cross-neutralizing DENV2 and DENV3. This presumptively is due to antibodies generated through epitope-decreasing immunization having greater binding capacity for EDIII domains.

The immunoglobulin repertoire sequencing results revealed that successive boost with epitope-decreased antigens generated similar Ig diversity as repeated immunization strategy. This suggest that repeated homologous immunization does not effectively increase Ig diversity as not all exposed surfaces of the DENV1 virus are antigenic epitopes. Consequently, epitope-decreased sequential immunization not only did not lead to a loss of Ig diversity, but also help to navigate T and B cells to focus on the conservative epitopes.

The sequential immunization approach presumptively can educate the memory cell to recognize homological domains that have a high probability of harboring conserved epitopes (11). Through stepwise boosts with antigens of decreasing epitope complexity, shared domains are emphasized thereby allowing the development of cross-reactivity. Sequential immunization serves to train the memory immune response to concentrate on “familiar” domains that already existed in the memory subset, thus directing antibody evolution. On the other hand, the consistent immunogens used in repeated immunization may burden the naïve or memory cells due to antigenic variation and frustrate memory maturation impeding cross-reactive Ab formation. In a realistic way, the principle of sequential immunization generally aligns with the reality for individuals living in dengue endemic areas, whose immune responses may become protective after multiple heterotypic exposures. Moreover, through this specific epitope decreasing approach, we were able to find a similar affect based on the use of sequential immunization but avoid the potential side effect of vaccine-induced ADE, which will pave the way for a safe and effective use of the vaccine and to combat the virus.

Finally, this study sheds light on how we can manipulate the immune system and supervise it to focus immunity on specific conserved domains to achieve the goal of broad cross-reactivity.

## Data Availability Statement

The datasets presented in this study can be found in online repositories. The names of the repository/repositories and accession number(s) can be found in the article/supplementary material.

## Ethics Statement

The animal study was reviewed and approved by National University of Singapore (NUS) Research Ethics Committee under Protocol R13-6157.

## Author Contributions

JH and JC designed this study and drafted the manuscript. WY drafted and revised the manuscript. JH, HL and LW conducted all assays.

## Funding

This work was supported by the National Research Foundation of Singapore through the Singapore–MIT Alliance for Research and Technology’s (SMART) Interdisciplinary Research Group in Infectious Disease Research Program.

## Conflict of Interest

The authors declare that the research was conducted in the absence of any commercial or financial relationships that could be construed as a potential conflict of interest.
